# Tyrosinase Inhibitors from the Stems of *Streblus Ilicifolius*

**DOI:** 10.1155/2021/5561176

**Published:** 2021-07-01

**Authors:** Nhan T. Nguyen, Phu H. Dang, Hai X. Nguyen, Truong N. V. Do, Tho H. Le, Tuyen Q. H. Le, Mai T. T. Nguyen

**Affiliations:** ^1^Faculty of Chemistry, University of Science, 227 Nguyen Van Cu Street, Ward 4, District 5, Ho Chi Minh City, Vietnam; ^2^Vietnam National University, Quarter 6, Linh Trung Ward, Thu Duc District, Ho Chi Minh City, Vietnam; ^3^Cancer Research Laboratory, University of Science, 227 Nguyen Van Cu Street, District 5, Ho Chi Minh City, Vietnam

## Abstract

Two new stilbene derivatives, named strebluses C and D, were isolated from the EtOAc-soluble fraction of the stems of *Streblus ilicifolius* (Moraceae). Its absolute configuration was elucidated based on NMR spectroscopic data interpretation and optical rotation calculation. Streblus C possesses strong tyrosinase inhibitory activity with an IC_50_ value of 0.01 *μ*M. Docking studies of **1** and **2** with *oxy*-tyrosinase were carried out to analyze their interactions. The analysis of the docked poses confirmed that **1** showed better binding affinity for *oxy*-tyrosinase than that of **2**.

## 1. Introduction

Tyrosinase (EC 1.14.18.1), which is a binuclear copper-containing monooxygenase, is a key enzyme in the oxidation of phenol to the corresponding *o*-quinone [1, 2]. It plays a main factor causing freckles, brown age spots, and melasma. Some commercial compounds, such as hydroquinone, arbutin, kojic acid, azelaic acid, l-ascorbic acid, ellagic acid, and tranexamic acid, were reported as the well-known tyrosinase inhibitors. These compounds have been used as skin whitening agents in cosmetic products, but they have certain drawbacks [3]. Thus, the finding of the new efficient and safe tyrosinase inhibitors is necessary for antihyperpigmentation product development.

*Streblus ilicifolius* (Vidal) Corner, which belongs to Moraceae family, was found and cultivated in Vietnam. Its barks have been traditionally used as an antipimple medicine. In a few published studies, some phenolic compounds have been reported in this plant [4–7]. In our continued studies on the screening of medicinal plants for tyrosinase inhibitory activity [8–12], it was found that a MeOH-soluble extract of the stems of *Streblus ilicifolius* showed a strong inhibitory effect, with an IC_50_ value of 0.63 *μ*g·mL^−1^. Thus, our study on chemical constituents of the stems of *S. ilicifolius* was carried out, leading to the isolation of two undescribed stilbene derivatives, strebluses C (**1**) and D (**2**). Compound **1** showed a strong tyrosinase inhibitory activity with an IC_50_ value of 0.01 *μ*M, which makes it 4400 times more potent than that of kojic acid (IC_50_, 44.6 *μ*M). In addition, molecular docking studies of **1** and **2** with the *oxy*-form of the copper-bound *Streptomyces castaneoglobisporus* tyrosinase were performed.

## 2. Materials and Methods

### 2.1. General Experimental Procedures

Optical values were measured on a Shimadzu UV-1800 spectrophotometer (Shimadzu Pte., Ltd., Singapore). IR spectra were measured with a Shimadzu IR-408 infrared spectrometer (Shimadzu Pte., Ltd., Singapore). NMR spectra were acquired on a Bruker Avance III 500 spectrometer (Bruker BioSpin AG, Bangkok, Thailand). Chemical shifts are expressed as *δ* values. HRESIMS data were acquired on Bruker micrOTOF-QII mass spectrometer (Bruker Singapore Pte., Ltd., Singapore). Column chromatography was carried out using silica gel 60, 0.06–0.2 mm (Scharlau, Barcelona, Spain), and LiChroprep RP-18, 40−63 *μ*m (Merck KGaA, Darmstadt, Germany). Kieselgel 60 F_254_ or RP-18 F_254_ plates for TLC were purchased from Merck (Merck KGaA, Darmstadt, Germany). Tyrosinase (EC 1.14.18.1) from mushroom (3933 U·mL^−1^) and l-dihydroxyphenylalanine (l-DOPA) were obtained from Sigma-Aldrich (Sigma-Aldrich Pte Ltd, Singapore). Other chemicals were of the highest grade available.

### 2.2. Plant Material

The stems of *Streblus ilicifolius* were collected at Hoai Nhon District, Binh Dinh Province, Vietnam, in October 2017. The plant was identified by Dr. rer. nat. Anh Tuan Dang-Le, Faculty of Biology and Biotechnology, University of Science, Ho Chi Minh City, Vietnam. A voucher sample (MCE0052) has been deposited at the Department of Medicinal Chemistry, Faculty of Chemistry, University of Science, Ho Chi Minh City, Vietnam.

#### 2.2.1. Extraction and Isolation

The dried powdered stems of *S. ilicifolius* (7.0 kg) were exhaustively extracted in a Soxhlet extractor with *n*-hexane, EtOAc, and MeOH to yield *n*-hexane-(64.8 g), EtOAc-(117.2 g), and MeOH-(378.0 g) soluble fractions, respectively. The EtOAc-soluble fraction was chromatographed by silica gel column chromatography (15 × 150 cm) and eluted with MeOH–CHCl_3_ (v/v, 0 : 100 ⟶ 100 : 0) mixtures to afford 18 fractions (Fr.1−Fr.18). Fraction Fr.8 (0.8 g) was separated by silica gel column chromatography with MeOH–CHCl_3_ (v/v, 0 : 100 ⟶ 30 : 70) mixtures to obtain six subfractions (Fr.8.1−Fr.8.6). Subfraction Fr.8.1 (34.8 mg) was loaded onto a silica gel column and eluted with EtOAc−CHCl_3_ mixtures (v/v, 0 : 100 ⟶ 100 : 0) and then purified by preparative TLC with an EtOAc−CHCl_3_ mixture (v/v, 15 : 85) to afford compound **2** (2.0 mg). Fraction Fr.14 (19.6 g) was subjected to further silica gel column chromatography and was eluted with CHCl_3_–*n*-hexane (v/v, 0 : 100 ⟶ 100 : 0) mixtures to yield 11 subfractions (Fr.14.1−Fr.14.11). Subfraction Fr.14.2 (69.0 mg) was chromatographed over a silica gel column with CHCl_3_–*n*-hexane (v/v, 0 : 100 ⟶ 100 : 0) mixtures to obtain two subfractions (Fr.14.2.1 and Fr.14.2.2). Subfraction Fr.14.2.2 (28.1 mg) was again chromatographed with CHCl_3_–*n*-hexane (v/v, 0 : 100 ⟶ 100 : 0) mixtures to give four subfractions (Fr.14.2.2.1−Fr.14.2.2.4). Subfraction Fr.14.2.2.3 (8.5 mg) was purified by preparative TLC with EtOAc−*n*-hexane (v/v, 20 : 80) mixture to afford **1** (3.9 mg).

#### 2.2.2. Streblus C (**1**)

Yellow, amorphous powder; ^1^H and ^13^C NMR (500 MHz, acetone-*d*_6_, see Table 1 and Figures S2–S7); HRESIMS *m/z* 393.1704 [M + Na]^+^ (Figure S8) (calcd for C_22_H_26_O_5_Na, 393.1678).

#### 2.2.3. Streblus D (**2**) (Figures S9–S15)

Yellow, amorphous powder; ^1^H and ^13^C NMR (500 MHz, acetone-*d*_6_, see Table 1 and Figures S8–S14); HRESIMS *m/z* 351.1224 [M + Na]^+^ (Figure S15) (calcd for C_19_H_20_O_5_Na, 351.1208).

### 2.3. Tyrosinase Inhibitory Assay

All pure compounds were dissolved in DMSO and tested at concentrations ranging from 0.01 to 100 *μ*M. Assay mixtures in 0.1 M phosphate buffer pH 6.8 were prepared immediately before use, consisting of 100 *μ*L of tyrosinase solution (15 U/mL) and 1900 *μ*L of test solution. These mixtures were preincubated at 32°C for 30 min, followed by addition of 1000 *μ*L of l-DOPA 1.5 mM in pH 6.8 phosphate buffer, and incubated at 32°C for 7 min. The absorbance (*A*) at 475 nm was acquired on Shimadzu UV-1800 spectrophotometer. The inhibitory percentage (*I*%) was calculated according to the formula: *I*% = [(*A*_control_ − *A*_sample_)/*A*_control_] × 100%. Data were represented as means ± standard error (*n* = 3). The IC_50_ values were determined by using GraphPad Prism software with multivariate nonlinear regression and *R*^2^ > 0.9. Kojic acid was used as positive control.

### 2.4. HPLC Data of the EtOAc-Soluble Fraction from *S. ilicifolius*

The concentrations of the EtOAc-soluble fraction and streblus C (**1**) were approximately 12,000 ppm and 200 ppm, respectively. The detection wavelength was set at 385 nm. An Agilent Zorbax SB-C18 column (150 × 4.6 × 5 mm) was used with a flow rate of 1 mL/min. The injection volume was 10 Μl, and the column temperature was maintained at 30°C. The mixtures of water and ACN were used as the mobile phase with gradient elution (20 ⟶ 40% ACN for 30 min).

### 2.5. Optical Rotation Calculation

The conformational searches were performed on Spartan'18 (Wave function, Inc., Irvine, USA) by using Merck molecular force field (MMFF). All conformers with Boltzmann weight ˃10% were optimized using *DFT* method at the B3LYP/6-31G∗ level in the gas phase, to give the preferred conformers with the Boltzmann weight >90%. The optical rotation calculations at sodium D line frequency were carried out using the B3LYP functional and the 6-311++G(2d, 2p) basis set in IEFPCM solvation model for methanol. These calculations were performed on Gaussian 09 (Gaussian, Inc., Wallingford, USA). The calculated optical rotation values were expressed as Boltzmann-weighted average of all output data.

### 2.6. Molecular Docking

Docking studies of **1** and **2**, positive reference (kojic acid), and decoy (hypoxanthine) were performed with Molecular Operating Environment 2019 (MOE 2019.0102) suite (Chemical Computing Group ULC, Montreal, Canada). The structures of these compounds were constructed by using the Builder module. Subsequently, all compounds were minimized up to 0.0001 gradients using the Amber12: EHT force field. The crystal structure of the *oxy*-tyrosinase was taken from the Protein Data Bank (PDB ID : 1WX2). The caddie protein (ORF378) and water molecules were removed. The enzyme structure was prepared using the QuickPrep module. The binding site was determined based on the Propensity for Ligand Binding (PLB) score in the Site Finder module. The molecular docking was performed by Dock Module, using Triangle Matcher Placement, Induced Fit Refinement, London Dg, and GBVI/WSA dG scoring methods. Five top poses showed up based on the negative binding free energy value (*S* value). The best pose was selected to analyze the receptor–ligand interactions by using BIOVIA Discovery Studio Visualizer 2016 (Dassault Systèmes Americas Corp., Waltham, USA).

## 3. Results and Discussion

### 3.1. Extraction and Isolation

**Figure 1:**
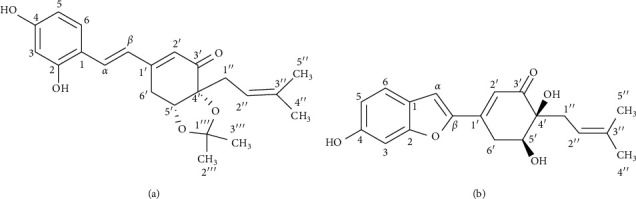
Structure of compounds **1** and **2**.

The dried powdered stems of *S. ilicifolius* were exhaustively extracted in a Soxhlet extractor with *n*-hexane, EtOAc, and MeOH to yield the corresponding fractions. The EtOAc-soluble fraction was repeatedly chromatographed using silica gel CC and preparative TLC to obtain two undescribed stilbene derivatives, strebluses C (**1**) and D (**2**) (Figure 1).

### 3.2. Structural Elucidation of Two New Isolated Compounds from *S. ilicifolius*

**Figure 2:**
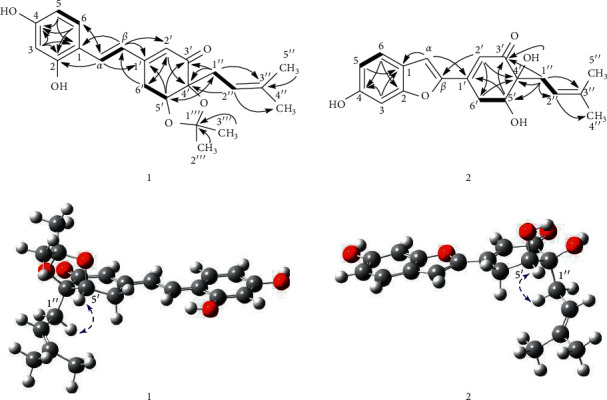
Significant HMBC (solid arrows) and NOESY (blue, dashed arrows) correlations observed for **1** and **2**.

Compound **1**, streblus C, showed a molecular formula to be C_22_H_26_O_5_ based on the HRESIMS sodium adduct ion at *m/z* 393.1704 [M + Na]^+^ (calcd for C_22_H_26_O_5_Na, 393.1678). The ^1^H NMR spectrum showed signals for a 1,2,4-trisubstituted aromatic ring [*δ*_H_ 7.48 (d, *J* = 8.5 Hz, H-6), 6.46 (d, *J* = 2.4 Hz, H-3), 6.42 (dd, *J* = 8.5, 2.4 Hz, H-5), two *trans*-coupling olefinic protons [*δ*_H_ 7.40 (d, *J* = 16.4 Hz, H-*α*), 7.01 (d, *J* = 16.4 Hz, H-*β*)], an *α*-olefinic proton of *α*, *β*-unsaturated carbonyl group [*δ*_H_ 5.97 (d, *J* = 2.0 Hz, H-2′)], an oxymethine proton [*δ*_H_ 4.48 (dd, *J* = 4.3, 1.6 Hz, H-5′)], a prenyl group [*δ*_H_ 2.54 (dd, *J* = 14.4, 8.0 Hz, H-1″a), 2.39 (dd, *J* = 14.4, 7.2 Hz, H-1″b), 5.18 (brs, H-2″), 1.68 (s, H_3_-4″), 1.63 (s, H_3_-5″)], two methyl groups [*δ*_H_ 1.32 (s, H_3_-2‴), 1.18 (s, H_3_-3‴)], and a methylene group [*δ*_H_ 3.19 (dd, *J* = 18.8, 1.6 Hz, H_2_-6′a), 2.82 (ddd, *J* = 18.8, 4.3, 2.0 Hz, H_2_-6′b)]. The ^13^C NMR data (Table 1) exhibited resonances for a keto-carbonyl (*δ*_C_ 199.2), six aromatic carbons and six olefinic carbons [*δ*_C_ 103.6–160.8], an acetonide group [*δ*_C_ 108.0, 27.8, 26.8], two oxygenated carbons [*δ*_C_ 82.8, 77.3], two methylene carbons [*δ*_C_ 33.0, 27.4], and two methyl carbons [*δ*_C_ 26.0, 18.1]. The HMBC correlations (Figure 2) from H-3 to C-1, C-2, and C-4, from H-5 to C-1 and C-4, from H-6 to C-2 and C-4, from H-*α* to C-2 and C-6, and from H-*β* to C-1, indicated that two hydroxy groups and C*α*-C*β* double bond located at C-2, C-4, and C-1, respectively, of the 1,2,4-trisubstituted aromatic ring. The presence of the cyclohex-2-en-1-one 5,6-acetonide moiety in **1** was established based on the observed HMBC correlations. The HMBC correlations from H-*α* to C-1′ and from H-*β* to C-1′ and C-2′ were supportive of the C*β*-C1′ linkage. In addition, the prenyl group was determined to be located at C-4′ by the HMBC correlations from H-1″ to C=O, C-4′, and C-5′ and from H-5′ to C-1″. Therefore, **1** was suggested to be a prenylated stilbene-like compound. The difference in chemical shifts of the methyl groups of the dimethylacetonide moiety in **1** is 0.14 ppm, which established the presence of the *cis*-acetonide [13]. Moreover, it was unambiguously confirmed based on the NOESY correlation between H-5′ and H_2_-1″ (Figure 2). The preferred conformations of the *cis*-(*R*, *R*)-acetonide **1** were generated by the MM2 calculation using MMFF94 force field [14]. These conformers were reoptimized by DFT-B3LYP method using basis set 6-31G∗, to obtain the most preferred conformer with 92.8% Boltzmann distribution (Table S1). The optical rotation value at sodium D line frequency was computed using B3LYP/6-311++G(2d, 2p) level with IEFPCM solvent model for methanol. The large basis set with diffuse functions such as 6-311++G(2d, 2p) was applied to give very consistent results [15, 16]. The calculated [*α*]_D_ value of (*R*,*R*)-acetonide **1** was −102.36, compared with its experimental value [*α*]_D_: −101.7 (*c* 0.023, MeOH). Thus, a (*R*, *R*) absolute configuration was concluded for streblus C (**1**).

A careful HPLC analysis of the EtOAc-soluble fraction was accomplished, which revealed a peak at *t*_*R*_ 20.766 min in the chromatogram in accord with that of **1** (*t*_*R*_ 20.800 min) (Figure S1). Thus, the presence of **1** in the EtOAc-soluble fraction from *S. ilicifolius* was confirmed, and the possibility of **1** being artifact could be ignored.

Compound **2**, streblus D, showed a molecular formula to be C_19_H_20_O_5_ based on the HRESIMS sodium adduct ion at *m/z* 351.1224 [M + Na]^+^ (calcd for C_19_H_20_O_5_Na, 351.1208). The ^1^H and ^13^C NMR data of **2** (Table 1) resembled those of **1**, except for the presence of the singlet olefinic proton at *δ*_H_ 7.31 instead of two *trans*-coupling olefinic protons in **1** and disappearance of the acetonide group. Based on the ^13^C NMR data and observed HMBC correlations for **2** (Figure 2), the structure of **2** was assigned as a benzofuran-type stilbene. The NOESY correlations between H-5′ and H_2_-1″ indicated the presence of the *cis*-diol configuration. The ^3^*J*_H-5′/H-6′_ coupling constants were 5.9 and 2.7 Hz, to suggest the equatorial configuration of H-5′ [17], which was supportive of the (*R*,*R*) or (*S*,*S*) absolute configurations for **2**. The conformational search for (*R*,*R*)-**2** was generated and optimized to obtain six conformers with total Boltzmann weight >90% (Table S1). The Boltzmann-weighted calculated [*α*]_D_ value of (*R*,*R*)-**2** was +301.74, compared with its experimental value [*α*]_D_: −228.9 (*c* 0.002, MeOH). Thus, a (*S*, *S*) absolute configuration was concluded for streblus D (**2**).

### 3.3. Tyrosinase Inhibitory Activity of Isolated Compounds from S. ilicifolius

Compounds **1** and **2** were tested for their tyrosinase inhibitory activities [18]. Kojic acid, a purported skin lightening agent, was used as a positive control. Streblus C (**1**) exhibited remarkable inhibitory effect with an IC_50_ value of 0.01 *μ*M, which was 4400 times more potent than that of kojic acid (IC_50_, 44.6 *μ*M). Meanwhile, streblus D (**2**) was inactive with an IC_50_ value > 100 *μ*M. These results were consistent with a previous report on the structure–activity relationships of stilbene derivatives. Compound **1** having 2,4-resorcinol subunit contributed the most to inhibitory activity [19]. In addition, the 2-arylbenzofuran derivatives showed lower tyrosinase inhibitory activities than the corresponding stilbene derivatives, suggesting that the formation of the five-membered ring led to the loss of inhibitory activity [20].

### 3.4. Docking Studies of Compounds **1** and **2**

Tyrosinase is an oxidase, which is represented as one of four possible forms (*deoxy*-, *oxy*-, *met*-, and *deact*- forms) [21]. *Oxy*-tyrosinase form oxidizes both phenols and catechols to *o*-quinones. Herein, mushroom tyrosinase (EC 1.14.18.1) plays the same role with respect to *oxy*-tyrosinase form. Two bound Cu^2+^ ions bind to six histidine residues, and the peroxide group is in the binding site of *oxy*-tyrosinase, which has a role in the catalytic oxidation [22]. To explore the strong inhibitory activity of **1** against tyrosinase, the molecular docking studies of **1** and **2**, respectively, with *oxy*-tyrosinase (PDB ID : 1WX2) were carried out [23].

The docking studies were performed with MOE. The top-ranked pose with the highest negative binding free energy value (*S* value) was selected for further interaction analysis with Discovery Studio Visualizer. Following our previous *in silico* study on tyrosinase inhibition, this docking procedure was already validated based on the docking results of the positive control (kojic acid) and the decoy (hypoxanthine) [12].

**Figure 3:**
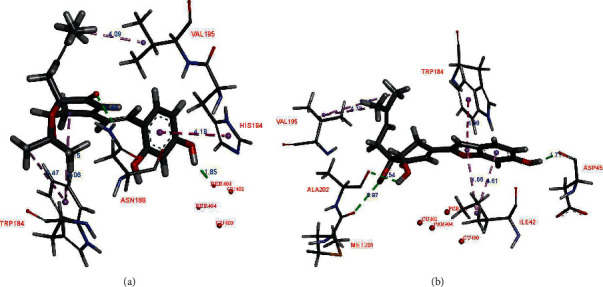
Docked pose of best ranked docking score of compounds **1** (a) and **2** (b).

In the binding site, compound **1** showed the H-donor interaction between the C-4 hydroxy group and peroxide bridge PER404, presenting the distances of 1.85 Å. The C-3′ carbonyl group formed the H-acceptor interaction with ASN188 residue (Figure 3). The aromatic ring exhibited the *π-π* stacking interaction with HIS194 residue localized in the active pocket. In addition, two methyls of the acetonide group showed the *π*-*σ* interactions with TRP184 residue. Compound **2** did not show any interaction with the catalytic site (i.e., Cu^2+^ ions and peroxide bridge), whereas kojic acid showed the interactions with a Cu^2+^ ion, HIS194, and THR203 residues in the binding site. Three hydroxy groups of **2** interacted with ASP45, ALA202, and MET201 residues via the H-donor bonding. The furan ring formed the *π-π* and *π*-*σ* interactions with TRP184 and ILE42 residues, respectively. The *S* values and these interactions suggested that **1** showed high binding affinity for *oxy*-tyrosinase than that of **2** (Table 2). This result confirmed that the formation of furan ring in **2** led to the loss of inhibitory activity.

## 4. Conclusions

Two new stilbene derivatives were isolated from the stems of *S. ilicifolius*. Their structures were elucidated based on the NMR spectroscopic interpretation and optical rotation calculation. Compound **1** was found to possess strong tyrosinase inhibitory activity with an IC_50_ value of 0.01 *μ*M. Binding interaction analyses between the isolated compounds (**1** and **2**) and *oxy*-tyrosinase active site have been performed.

## Figures and Tables

**Table 1:** ^1^H (500 MHz) and ^13^C (125 MHz) NMR data (acetone-*d*_6_) for compounds **1** and **2**.

Position	**1**		**2**	
	*δ*_C_, type C	*δ*_H_ (*J*, Hz)	*δ*_C_, type C	*δ*_H_ (*J*, Hz)
1	116.3, C		122.0, C	
2	158.3, C		158.0, C	
3	103.6, CH	6.46, d (2.4)	98.3, CH	7.00, d (2.1)
4	160.8, C		158.9, C	
5	108.9, CH	6.42, dd (8.5, 2.4)	114.3, CH	6.87, dd (8.5, 2.1)
6	129.6, CH	7.48, d (8.5)	123.4, CH	7.52, d (8.5)
1′	153.9, C		143.6, C	
2′	125.0, CH	5.97, d (2.0)	118.7, CH	6.48, d (2.1)
3′	199.2, C=O		200.6, C=O	
4′	82.8, C		79.6, C	
5′	77.3, CH	4.48, dd (4.3, 1.6)	73.0, CH	4.27, dd (5.9, 2.7)
6′	27.4, CH_2_	3.19, dd (18.8, 1.6) 2.82, ddd (18.8, 4.3, 2.0)	32.0, CH_2_	3.15, ddd (18.5, 5.9, 2.1) 3.08, dd (18.5, 2.7)
*α*	132.3, CH	7.40, d (16.4)	110.7, CH	7.31, s
*β*	126.4, CH	7.01, d (16.4)	153.2, C	
1″	33.0, CH_2_	2.54, dd (14.4, 8.0) 2.39, dd (14.4, 7.2)	35.3, CH_2_	2.47, dd (14.9, 7.7) 2.40, dd (14.9, 7.0)
2″	118.1, CH	5.18, brs	118.8, CH	5.22, brt (7.3)
3″	136.0, C		135.0, C	
4″	26.0, CH_3_	1.68, s	26.1, CH_3_	1.67, s
5″	18.1, CH_3_	1.63, s	18.1, CH_3_	1.58, s
1‴	108.0, C			
2‴	27.8, CH_3_	1.32, s		
3‴	26.8, CH_3_	1.18, s		
4-OH				8.88, s
4′-OH				4.18, s
5′-OH				3.83, s

**Table 2:** Docking results of **1** and **2** with *oxy*-tyrosinase.

Compound	*oxy*-tyrosinase (1 WX2)			
	*S* values	Interactions	Targeting residues	Distance (Å)
**1**	–6.56	H-donor	PER404	1.85
		H-acceptor	ASN188	2.44
		*π*-*π*	HIS194	4.19
		*π*-*σ*	TRP184	5.15
				5.06
				5.47
		*σ*-*σ*	VAL195	4.09

**2**	–6.17	H-donor	ASP45	1.71
			ALA202	2.54
			MET201	2.97
		*π*-*π*	TRP184	4.98
		*π*-*σ*	ILE42	4.66
				4.61
		*σ*-*σ*	VAL195	4.73

Kojic acid^a^	–4.50	H-donor	THR203	2.04
		Metal-acceptor	CU401	2.92
		*π*-*π*	HIS194	4.30

^a^Positive control.

## Data Availability

The NMR data used to support the findings of this study are included within the supplementary information file.

## Supplementary Materials

Supplementary Materials Figure S1: HPLC chromatogram of **1** from the stems of *S. ilicifolius*. Table S1: conformational data. Figures S2–S15: copies of spectroscopic data for **1** and **2**.
