# Supplementary material for: Tyrosinase Inhibitors from the Stems of Streblus Ilicifolius
Source: Evid Based Complement Alternat Med. 2021 Jul 1;2021:5561176. doi: 10.1155/2021/5561176 (PMC8266447; doi:10.1155/2021/5561176)
Supplement: Supplementary Materials — Figure S1: HPLC chromatogram of 1 from the stems of S. ilicifolius. Table S1: conformational data. Figures S2–S15: copies of spectroscopic data for 1 and 2. [file 5561176.f1.docx]

*Supplementary data*

**Tyrosinase Inhibitors from the Stems of *Streblus ilicifolius***

Nhan T. Nguyen,^1,2,3^ Phu H. Dang,^1,2^ Hai X. Nguyen,^1,2^ Truong N. V. Do,^1,2^ Tho H. Le,^1,2^ Tuyen Q. H. Le,^1,2^ and Mai T. T. Nguyen*^,1,2,3^

^1^ Faculty of Chemistry, University of Science, 227 Nguyen Van Cu Street, Ward 4, District 5, Ho Chi Minh City, Vietnam

^2^ Vietnam National University, Quarter 6, Linh Trung Ward, Thu Duc District, Ho Chi Minh City, Vietnam

^3^ Cancer Research Laboratory, University of Science, 227 Nguyen Van Cu Street, District 5, Ho Chi Minh City, Vietnam

Mai T. T. Nguyen; [nttmai@hcmus.edu.vn](mailto:nttmai@hcmus.edu.vn)

**Abstract**

Two new stilbene derivatives, named strebluses C and D, were isolated from the EtOAc-soluble fraction of the stems of *Streblus ilicifolius* (Moraceae). Its absolute configuration was elucidated based on NMR spectroscopic data interpretation and optical rotation calculation. Streblus C possesses strong tyrosinase inhibitory activity with an IC_50_ value of 0.01 *µ*M. Docking studies of **1** and **2** with *oxy*-tyrosinase were carried out to analyze their interactions. The analysis of the docked poses confirmed that **1** showed better binding affinity for *oxy*-tyrosinase than that of **2**.

***Keywords:*** *Streblus ilicifolius*; Stilbene derivative; Tyrosinase inhibitory; Molecular docking.

**Table S1:** Energies and calculated [*α*]_D_ values of conformers of (*R*,*R*)-**1** (no. 1) and (*R*,*R*)-**2** (no. 2–7) at B3LYP/6-31G* in gas phase.

| no |  | Conformer |  | E (kJ/mol) |  | Boltzmann distribution (%) | Calculated [*α*]_D_ |
| --- | --- | --- | --- | --- | --- | --- | --- |
| 1 |  | 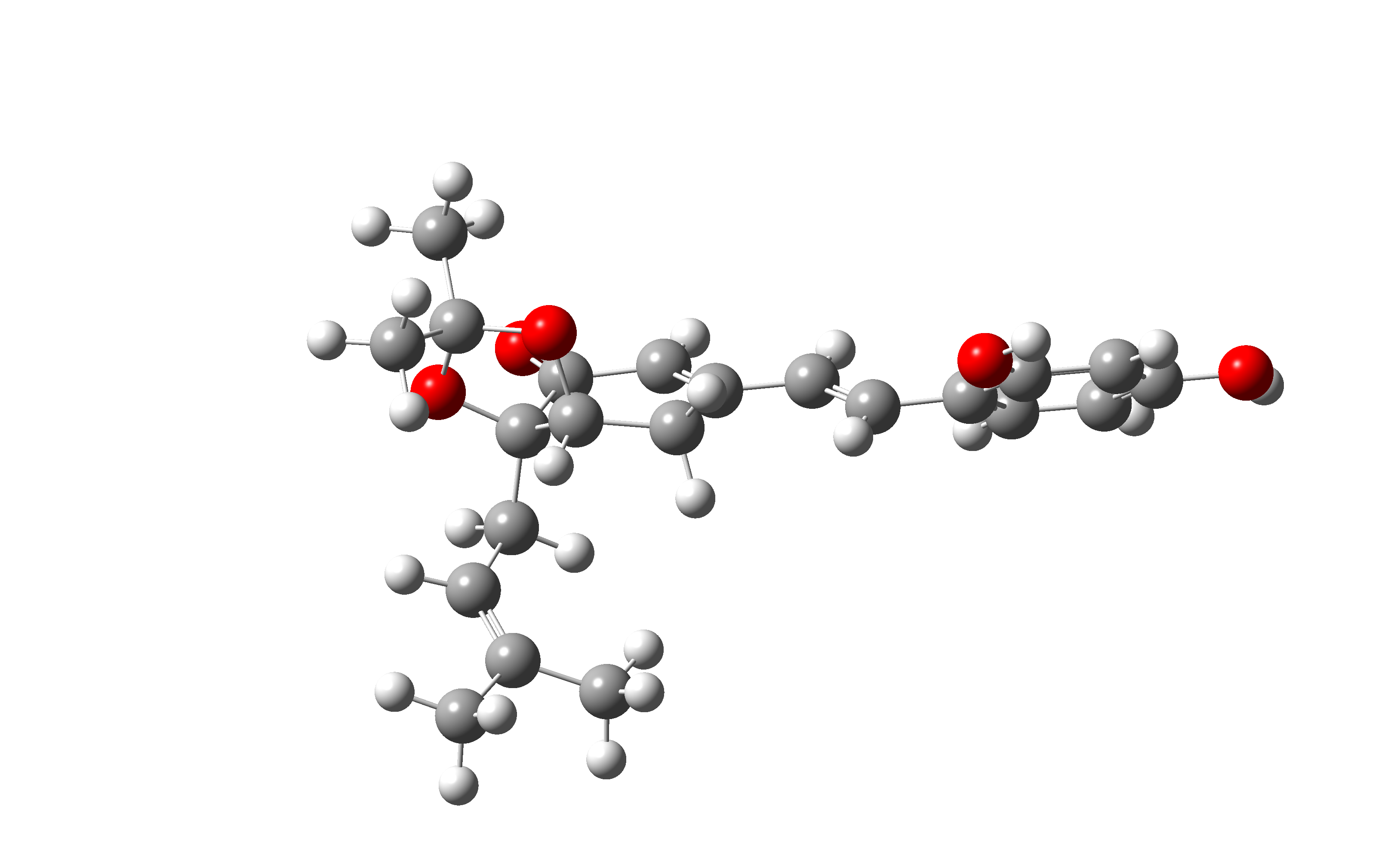 |  | −3,229,535.75 |  | 92.8 | −102.36 |
| 2 |  | 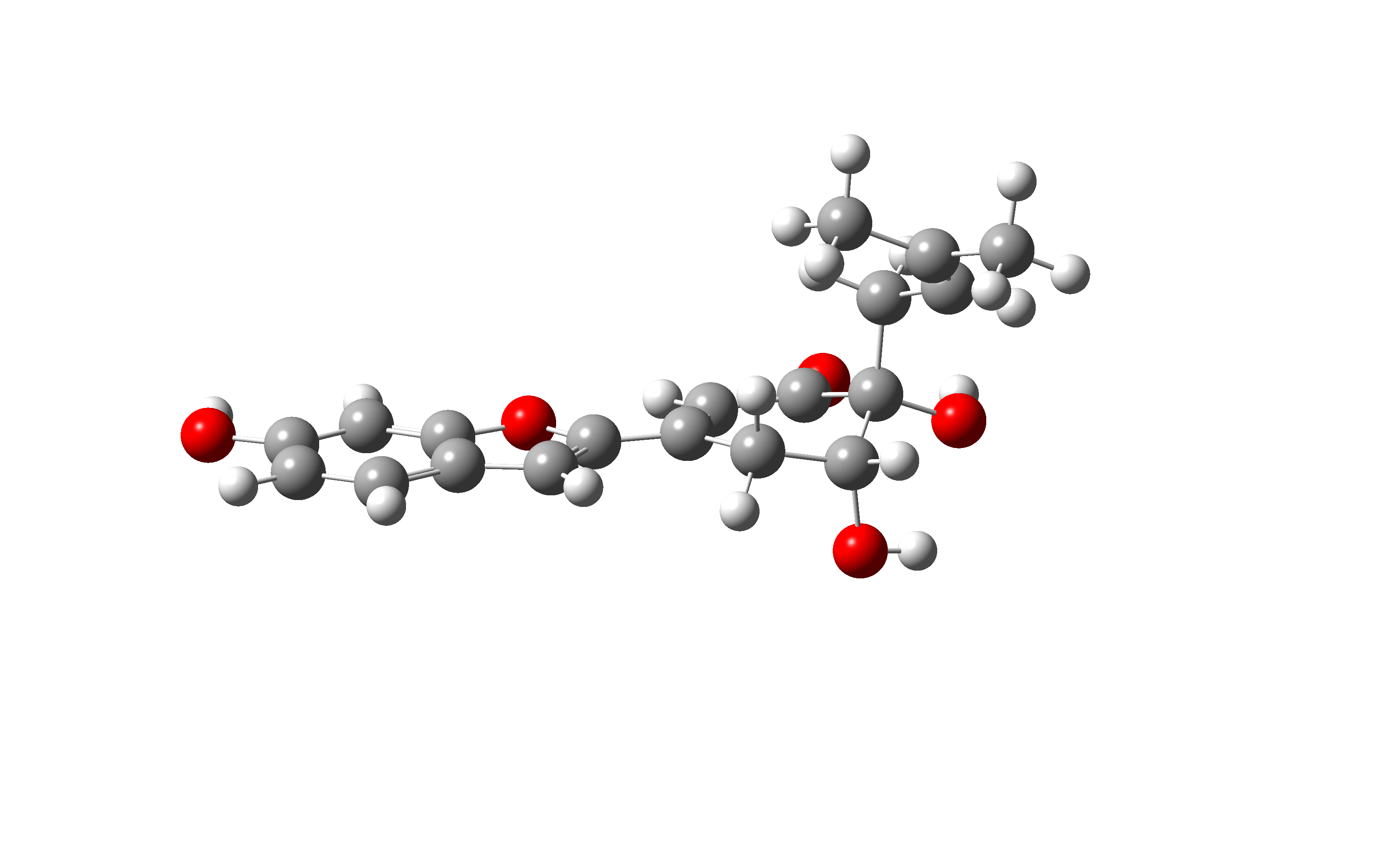 |  | −2,919,912.3 |  | 25.1 | +725.29 |
| 3 |  | 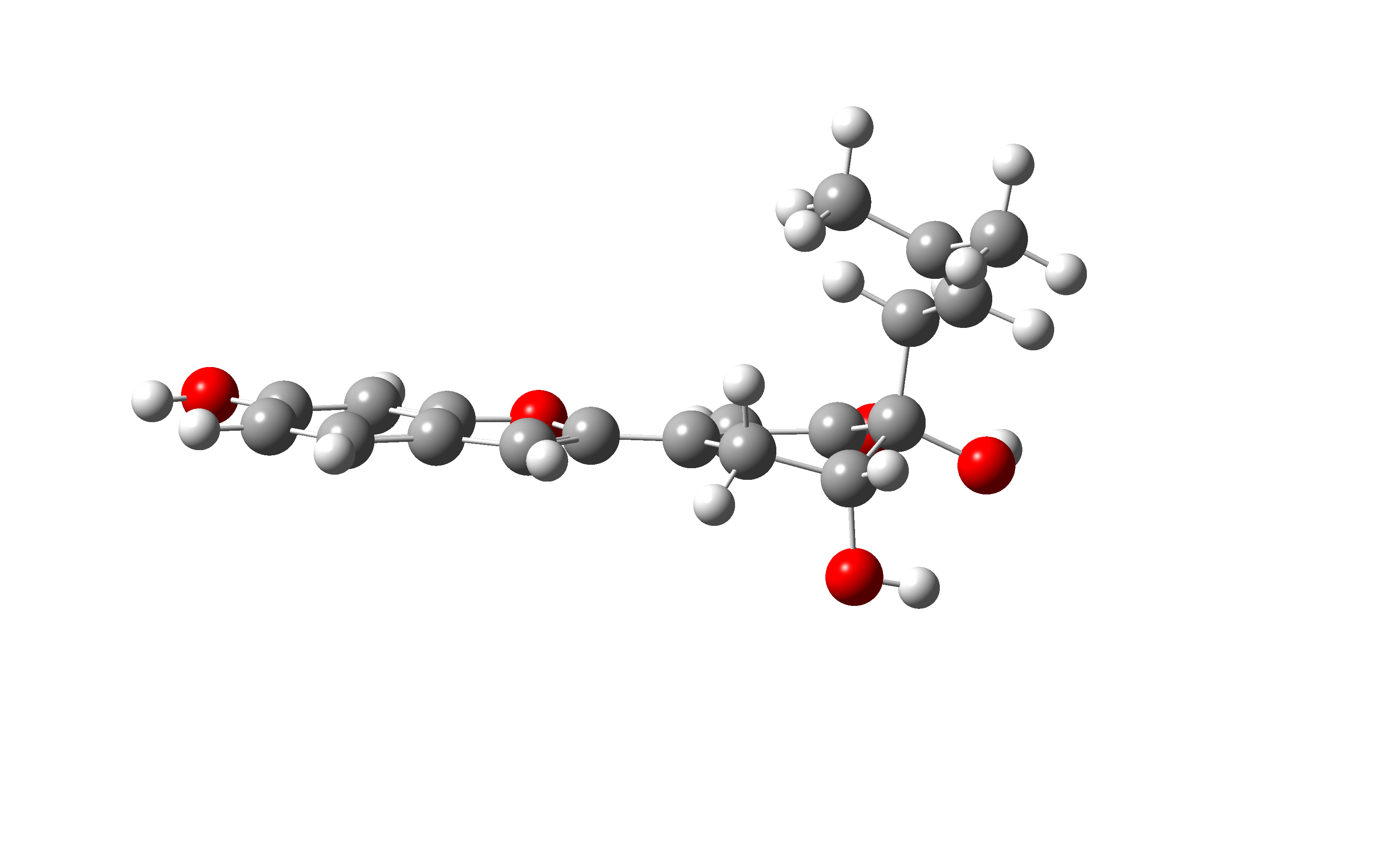 |  | −2,919,911.6 |  | 18.9 | +731.09 |
| 4 |  | 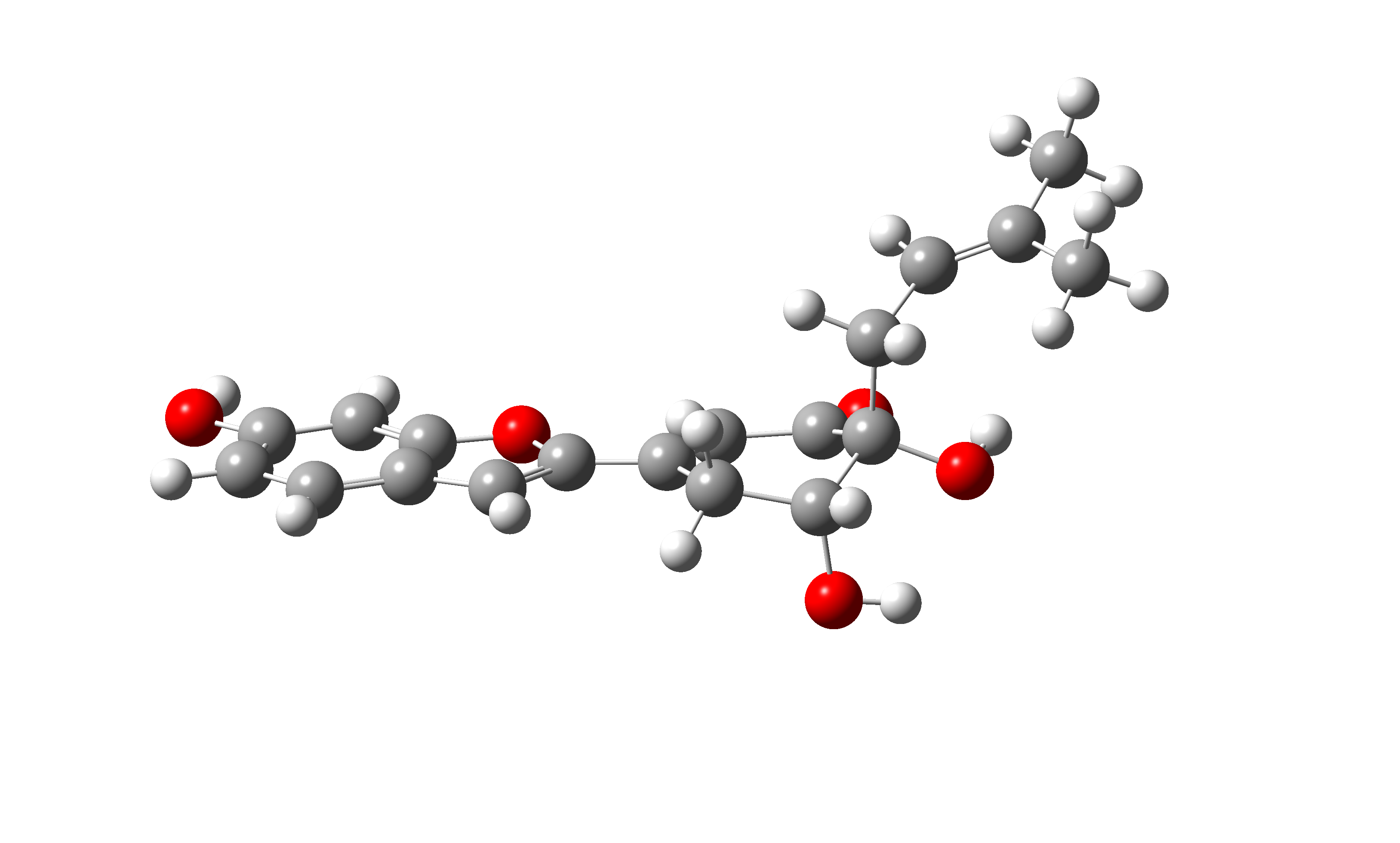 |  | −2,919,911.0 |  | 15.3 | +155.63 |
| 5 |  | 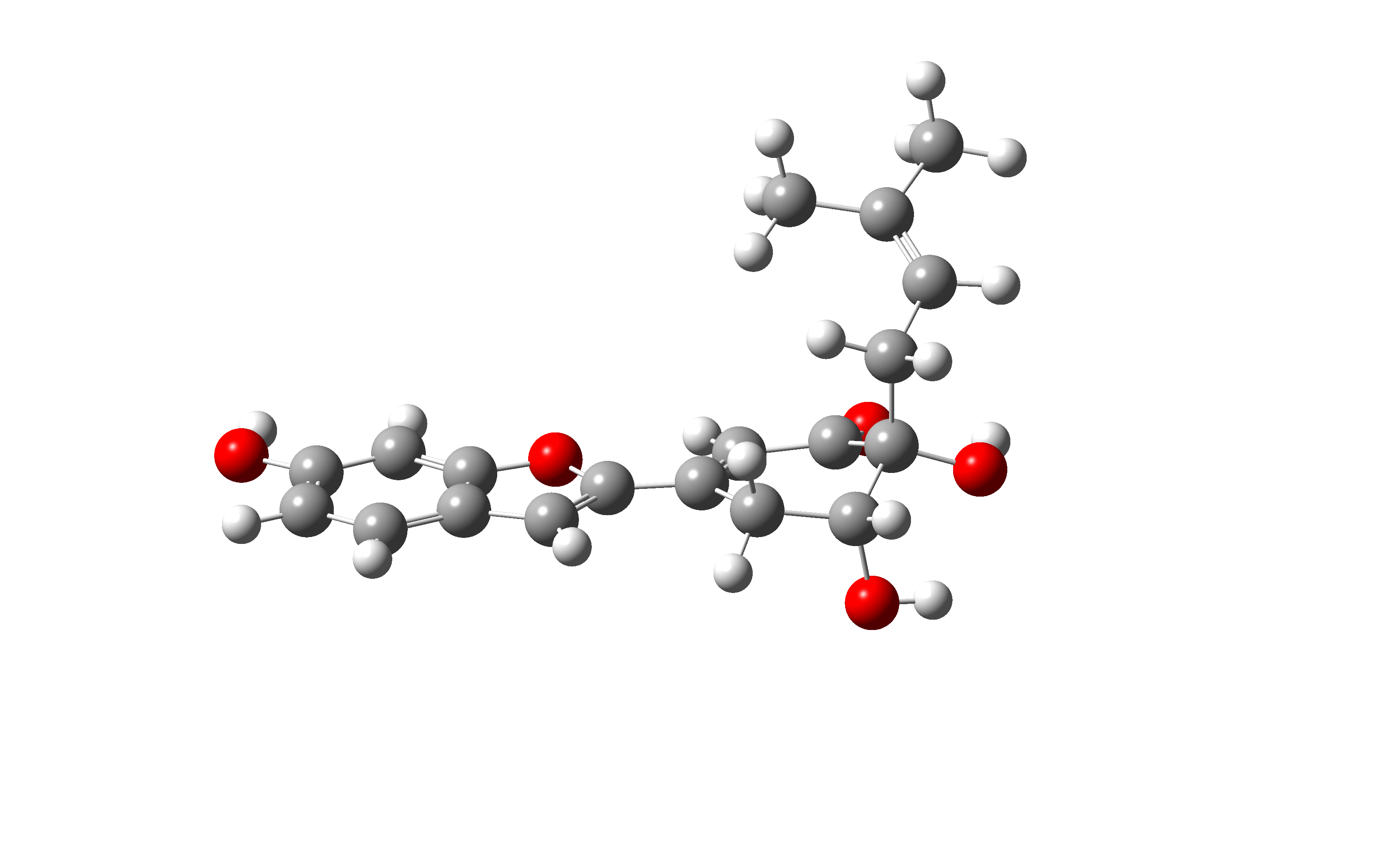 |  | −2,919,910.8 |  | 13.6 | −102.22 |
| 6 |  | 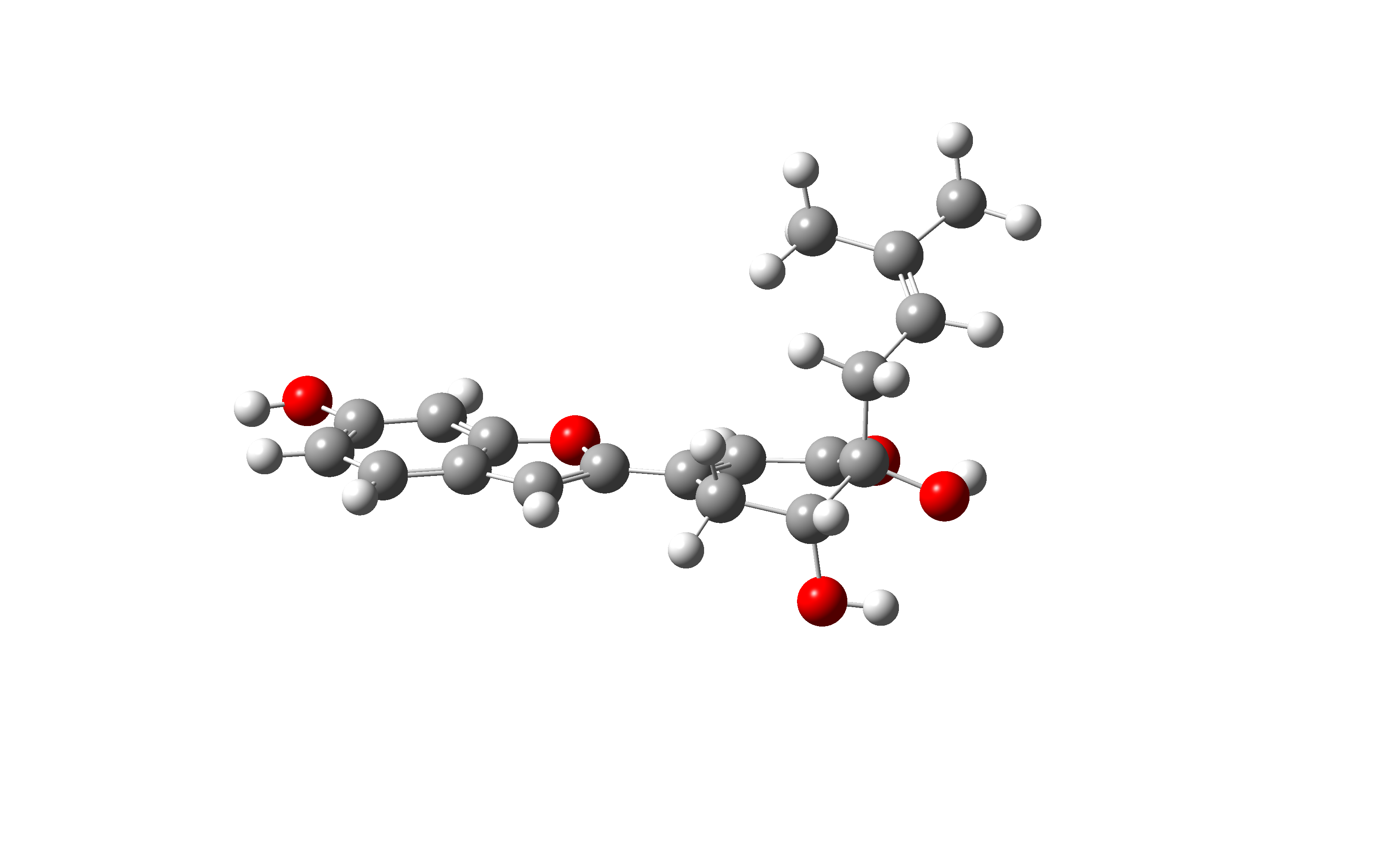 |  | −2,919,909.9 |  | 9.7 | −107.03 |
| 7 |  | 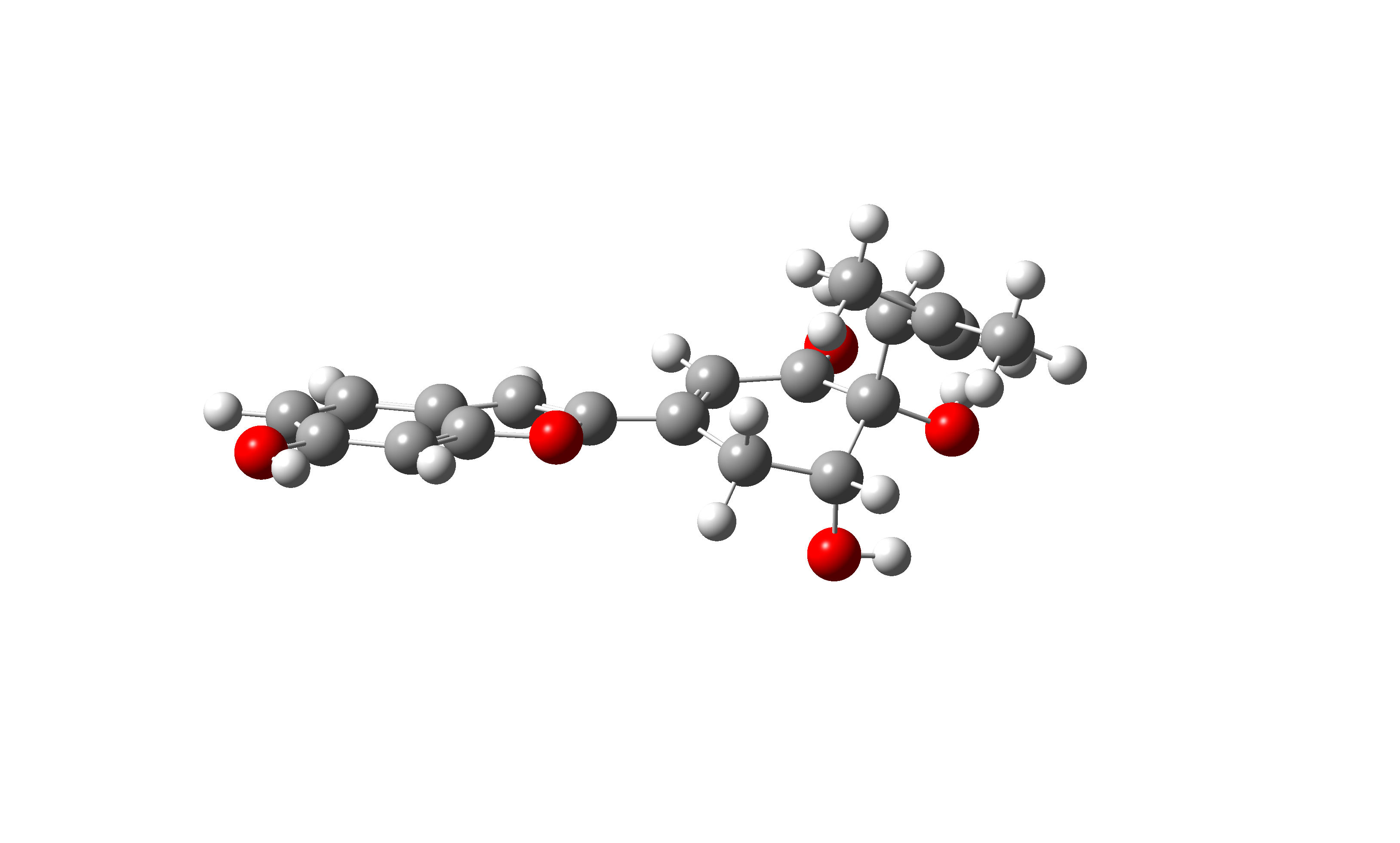 |  | −2,919,909.5 |  | 8.2 | −219.61 |

**Figure S1.** HPLC chromatogram of streblus C (red line) and the EtOAc-soluble fraction of *S. ilicifolius* (blue line)

**Figure S2.** ^1^H NMR spectrum of **1** (500 MHz, acetone-*d*_6_).

**Figure S3.** ^13^C NMR spectrum of **1** (125 MHz, acetone-*d*_6_).

**Figure S4.** HSQC NMR spectrum of **1**.

**Figure S5.** HMBC NMR spectrum of **1**.

**Figure S6.** COSY NMR spectrum of **1**.

**Figure S7.** NOESY NMR spectrum of **1**.

**Figure S8.** HRESIMS spectrum of **1**.

**Figure S9.** ^1^H NMR spectrum of **2** (500 MHz, acetone-*d*_6_).

**Figure S10.** ^13^C NMR spectrum of **2** (125 MHz, acetone-*d*_6_).

**Figure S11.** HSQC NMR spectrum of **2**.

**Figure S12.** HMBC NMR spectrum of **2**.

**Figure S13.** COSY NMR spectrum of **2**.

**Figure S14.** NOESY NMR spectrum of **2**.


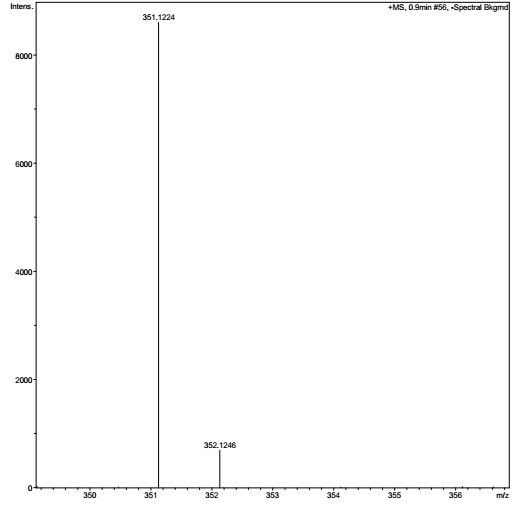


**Figure S15.** HRESIMS spectrum of **2**.
